# Improving the refractive outcomes of cataract surgery

**Published:** 2025-10-03

**Authors:** Thulasiraj Ravilla, Gladys Atto, John Buchan

**Affiliations:** 1 Director Operations: Aravind Eye Care System, India.; 2 Opthalmologist: Moroto Regional Referral Hospital, Moroto, Uganda.; 3 Programme Director: MSc Public Health for Eye Care, ICEH, London Schoolof Hygiene & Tropical Medicine and Clinical Lead: RCOphth National Ophthalmology Database Cataract Audit, Leeds, UK.

**Poor refractive outcomes form one of the main reasons for cataract surgery failing to provide patients with the vision they need to function well.**

**Figure:**
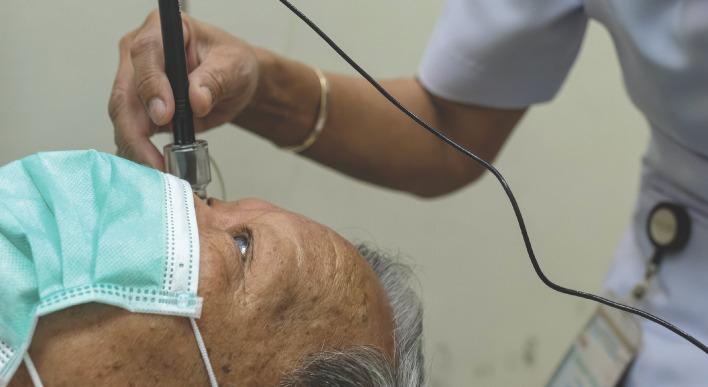
A nurse performs biometry on a cataract patient. LAO PDR

© THE FRED HOLLOWS FOUNDATION CC BY-NC-SA 4.0

Cataract is the leading cause of blindness worldwide, despite an effective cure having been available for several decades, in the form of cataract surgery.

## A focus on biometry and IOLs

One of the main reasons for cataract surgery failing to provide patients with the vision they need is poor refractive outcomes. In this issue, we will explore how refractive outcomes can be monitored and improved through good biometry, intraocular lens (IOL) selection, and auditing to improve biometry – all of which helps to minimise the number of people who remain limited in their visual function after undergoing cataract surgery. Our next issue will focus on surgical quality and safety, which is equally vital, and we will be covering patient-centred cataract services, including postoperative refraction, in early 2026.

Peter Drucker, a celebrated management guru, said, “If you can't measure it, you can't improve it.” This applies to cataract surgery outcomes as well. If we don't know what vision our cataract patients are left with after surgery, there is little scope for improving it. For individual surgeons and hospitals, this means seeing your patients postoperatively and collecting data on complication rates and visual outcomes. Historically, national level monitoring just involved asking, “What proportion of those who need cataract surgery have had surgery?” This was the **cataract surgical coverage** (CSC). Although helpful it did not provide any information about the quality and effectiveness of that operation from the patient's perspective.

## Effective cataract surgical coverage

The cataract outcome indicator now promoted by the World Health Organization (WHO) is **effective cataract** **surgical coverage (eCSC)**. This is the proportion of all those who had developed cataract (both those operated for cataract and those still in need of surgery) who have been operated on and who have a presenting visual acuity of 6/12 or better. (Note: presenting visual acuity is measured with whatever correction a person is currently using.) This effectively raises the standard from the previous threshold for “good” outcomes of 6/18 or better.^1^

The WHO have recommended a target for countries to increase eCSC by 30 percentage points by 2030. Population-based surveys conducted in 55 countries showed substantial variation in eCSC, ranging from 3.8% to 70.3%. The median eCSC was 24.8%, while the median CSC stood at 40.0%. The relative quality gap (the difference between 40.0% and 24.8%, expressed as a percentage of 40.0%) is 38%; this means that over one-third of operated patients did **not** achieve a good outcome of ≥ 6/12 presenting visual acuity. In settings where the relative quality gap exceeds 25%, it is recommended to prioritise quality improvement initiatives before scaling up surgical access or volume.^2^ The IAPB Vision Atlas provides estimates of CSC and eCSC for several ountries (see visionatlas.iapb.org).

In India, data from population-based surveys conducted in 31 districts reported an overall eCSC of 36.7% and a CSC of 57.3%, giving a relative quality gap of 35.9%. This indicates that, in more than one-third of operated patients, the presenting vision was below the threshold of 6/12.^3^ This gap was more pronounced among patients who underwent manual small incision cataract surgery (MSICS), at 39%, compared to 8% in those who had phacoemulsification.^4^ Nonetheless, MSICS is likely to continue to be the preferred technique as it doesn't need expensive equipment, with its accompanying maintenance challenges.

In one Liberian hospital-based study, at 4–11 weeks, good outcomes of 6/12 or better were reported in 38.6% of patients (uncorrected visual acuity) and 82.5% (best-corrected visual acuity).^5^ In an Indian hospital-based study with a much larger sample size of about 84,000 patients, it was found that 54.4% of operated eyes (71.2% of MSICS eyes and 21.0% of phacoemulsification eyes) showed a potential improvement of 2 lines or more of Snellen acuity with refraction.^6^ This evidence suggests that a lack of postoperative follow-up and refraction is a significant cause of the quality gap between CSC and eCSC. Attention should be paid to optimising the direct refractive outcomes of surgery, particularly in settings where the uptake of postoperative spectacles – including near vision spectacles for presbyopia – is low. However, even where biometry and lens choices have been optimised, effective postoperative refractive management will improve patients’ visual function and quality of life after cataract surgery.^7^

## Refractive aims: does one size fit all?

Targeting good distance vision is appropriate for most patients, and there is the expectation that those who need good near vision postoperatively will access reading glasses. However, there are settings in which long-term use of near vision spectacles is very limited following cataract surgery, despite affordable spectacles being made more accessible; this may be for cultural reasons, or where literacy levels amongst the elderly are low. For such patients, emmetropia may not be the best option and a low myopic aim would provide acceptable distance vision with more near functionality. There may be other patients who spend the majority of their time on near tasks, and who would prefer to prioritise getting good unaided near vision with a myopic postoperative refractive target.

There is no evidence currently available to tell us what refractive outcome provides patients with the best function, quality of life, or satisfaction with their surgery. The best refractive aim for maximised quality of life scores is likely to vary according to gender, age, and socioeconomic status as well as between and within countries, as patient preferences and visual demands vary according to circumstances. It is also possible that the best visual function in patients who are less likely to sustain spectacle use postoperatively would be to have one eye focused near emmetropia, with the other eye being left with a low myopic correction. For this reason, it is important that clinicians listen to patients in order to understand each individual's needs.

There is no point discussing targets, unless a service is achieving sufficient accuracy with biometry: the process of measuring the power of the cornea (keratometry) and length of the eyeball (axial length) and using this data to determine the ideal IOL power. Biometry for all patients undergoing cataract surgery is no longer considered optional by the majority of eye care professionals. Equally, if various powers of IOL are unavailable, efforts with biometry are wasted. We have therefore included articles on practical approaches to biometry in lower-resource settings, auditing to improve the outcomes of biometry, and IOL choice and management to improve availability. We also have an excellent case study demonstrating how biometry can improve outcomes in hospital and in outreach settings.

Good refractive outcomes and patient satisfaction with surgery is dependent upon the collective efforts of everyone involved in the patient journey, including the ophthalmologist, counsellor, biometry technician, supply-chain person, operating room team, and refractionist/optometrist. We aim to provide evidence-based recommendations and case studies for service improvement at each step in the process in this issue and our next three issues.

*Look out for our next three issues, covering the surgical quality and safety of cataract services; presbyopia; and patient-centred cataract care. Subscribe at* cehjournal.org/subscribeonline *to make sure you don't miss any!*
